# 

**Published:** 1930

**Authors:** 


### The Bristol Medico-Chirurgical Journal

A JOURNAL OF THE MEDICAL SCIENCES FOR THE
WEST OF ENGLAND AND SOUTH WALES.

PUBLISHED UNDER THE AUSPICES OF
*THE BRISTOL MEDICO-CHIRURGICAL SOCIETY.*

EDITOR:
J. A. NIXON, C.M.G., M.D., F.R.C.P.

WITH WHOM ARE ASSOCIATED

E. W. HEY GROVES, M.S., F.R.C.S., B.Sc.
E. WATSON-WILLIAMS, M.C., Ch.M., F.R.C.S.E.
A. E. ILES, O.B.E., M.B., B.S. Lond., F.R.C.S.
CAREY F. COOMBS, M.D., F.R.C.P., Assistant Editor.

EDITORIAL SECRETARY:
A. L. FLEMMING, M.B., B.Ch.

"Scire est nescire, nisi id me

Scire alius sciret."

*VOL. XLVII.*

BRISTOL: J. W. ARROWSMITH LTD.
London: J. W. Arrowsmith (London) Ltd.

R31
.B8

### Contents of Vol. XLVII.

| | Page |
|---|---|
| The Duty of Medicine to the Race. By C. Ferrier Walters, F.R.C.S. | 1 |
| Hospitals—Voluntary or Self-Supporting ? By R. J. A. Berry, M.D., F.R.C.S., F.R.S. .. .. .. .. .. .. .. | 19 |
| The Infective Factor in Thrombosis and Embolism. By Ambrose W. Owen, M.D. .. .. .. .. .. .. .. .. | 29 |
| Acute Intestinal Obstruction. By D. P. D. Wilkie, O.B.E., M.D., Ch.M., F.R.C.S., F.R.S.E. .. .. .. .. .. .. | 97 |
| Child Guidance :— | |
| I. Work in New York. By Mrs. Posthuma, M.B., Ch.B. .. | 111 |
| II. Clinics from the Brain Physiologist's Viewpoint. By R. J. A. Berry, M.D., F.R.C.S., F.R.S.E. .. .. .. | 120 |
| III. Work in Great Britain. By R. G. Gordon, M.D., D.Sc., F.R.C.P.E. .. .. .. .. .. .. .. | 126 |
| Stokes-Adams Syndrome produced by Digitalis in a Case of Auricular Fibrillation. By T. F. R. Hewer, M.D. .. .. .. .. | 135 |
| Dental Infections : their Diagnosis and Treatment. By G. F. Fawn, M.R.C.S., L.R.C.P., B.D.S., L.D.S. .. .. .. .. .. | 137 |
| Recent Dental Research. By R. H. McKeag, M.A., M.B., Ch.B. .. | 143 |
| Observations on Epilepsy. By J. V. Blachford, C.B.E., M.D. .. | 169 |
| Five Years' Experience of Surgery in Exophthalmic Goitre. By A. Rendle Short, B.Sc., M.D., B.S., F.R.C.S. .. .. .. | 185 |
| The Treatment of Graves' Disease. By C. E. K. Herapath, M.C., M.D. | 193 |
| Changes in the Spleen in Acute Pyogenic Infections. By T. F. R. Hewer, M.D. .. .. .. .. .. .. .. .. | 197 |
| A Case of von Recklinghausen's Disease (Multiple Neurofibromata) with Spontaneous Fractures. By L. P. Ashton .. .. .. | 219 |
| The Origin and Significance of Tuberculosis of the Tracheo-Bronchial Lymph Nodes of the Lung. By L. R. Jordan .. .. .. | 225 |
| The Long Fox Memorial Lecture : The Influence of Food on the Production and Prevention of Disease. By J. A. Nixon, C.M.G., M.D., F.R.C.P. .. .. .. .. .. .. .. .. | 255 |
| The Beddoe Memorial Lecture : Anthropology : Old and New. By Sir Arthur Keith, F.R.S. .. .. .. .. .. .. | 287 |
| Notes on the Treatment of Eclampsia. By G. H. Temple, M.B., C.M. | 307 |
| Five Cases of Injury to Main Vessels occurring in Civil Practice. By Robert V. Cooke, M.B., Ch.M., F.R.C.S. .. .. .. .. | 311 |
| Perforation in Paratyphoid B. By Frank Bodman, M.B., M.R.C.S., and Paul Bodman, M.B. .. .. .. .. .. .. | 317 |
| A Case of Typhoid Fever with Co-existent Bacillus Coli Infection. By J. Innes Noble, M.B., Ch.B., F.R.C.S. .. .. .. .. | 323 |
| Reviews of Books .. .. .. .. .. .. | 69, 149, 239, 331 |
| Editorial Notes .. .. .. .. .. .. | 76, 159, 248, 339 |
| Obituaries .. .. .. .. .. .. .. .. .. | 164, 342 |
| Meetings of Societies .. .. .. .. .. .. | 78, 165, 346 |
| The Medical Library of the University of Bristol | 84, 166, 252, 350 |
| Local Medical Notes .. .. .. .. | 87, 167, 254, 351 |

### The Medical Library of the University of Bristol,

WITH WHICH IS INCORPORATED

**The Library of the Bristol Medico-Chirurgical Society.**

---

*The following donations have been received since the publication of the List in December,* 1929.

*March,* 1930.

| | | |
|---|---|---|
| The Governor, Gold Coast Colony (1) | .. | 1 report. |
| Far Eastern Association of Tropical Medicine (2) | | 2 volumes. |
| Michell Clarke Memorial Fund (3) | .. .. | 41 ,, |
| Munro Smith Memorial Fund (4) | .. .. | 9 ,, |
| Rockefeller Foundation (5) | .. .. .. | 2 ,, |
| International Congress of Tropical Medicine (6) | | 2 ,, |

Unbound periodicals have also been received from Dr. Carey F. Coombs, Mr. Lacy Firth and Professor E. W. Hey Groves.

### THE ONE HUNDRED AND THIRTY-FIRST LIST OF BOOKS.

The figures in round brackets refer to the figures after the names of the donors. The books to which no such figures are attached have either been bought from the Library Fund or received through the *Journal.*

| | | | | |
|---|---|---|---|---|
| **Abel, A. L.** | *Oesophageal Obstruction* | | | 1929 |
| **Bainbridge and Menzies.** | *Essentials of Physiology* | 6th Ed. | (3) | 1929 |
| **Berkeley, C.** | *Handbook of Midwifery* | 7th Ed. | (3) | 1929 |
| **Berry, R. J. A.** | *Through the States with a Seeing Eye* | | | 1930 |
| **Bigger, J. W.** | *Handbook of Bacteriology* | 2nd Ed. | (3) | 1929 |
| **Boyd, W.** | *Surgical Pathology* | 2nd Ed. | (3) | 1929 |
| **Brain, W. R., and Strauss, E. B.** | *Recent advances in Neurology* | | (4) | 1929 |
| **Brock, A. J.** | *Greek Medicine* | | (3) | 1929 |
| **Burrell, A. S. T.** | *Recent advances in Pulmonary Tuberculosis* | | (3) | 1929 |
| **Cameron, H. C.** | *The Nervous Child* | | | 1929 |

| | | | | |
|---|---|---|---|---|
| **Chauvin, E.** | *Maladies des Vésicules Séminales* | | | 1930 |
| **Clark, W. M.** | *Determination of Hydrogen ions* | 3rd Ed. | (3) | 1928 |
| **Conybeare, J. J.** | *Textbook of Medicine* | | (3) | 1929 |
| **Crofton, W. M.** | *Outline of Endocrinology* | 2nd Ed. | (3) | 1929 |
| **Curschmann, H.** | *Endocrine Disorders* | | | 1929 |
| **Dew, H. R.** | *Hydatid Disease* | | (3) | 1928 |
| **Dible, J. H.** | *Recent Advances in Bacteriology* | | (4) | 1929 |
| **Dixon, W. E.** | *Manual of Pharmacology* | 7th Ed. | (3) | 1929 |
| **East, C. F. T. and Bain, G. W. C.** | *Recent advances in Cardiology* | | (3) | 1929 |
| **Forsdike, S.** | *Sterility in Women* | | (3) | 1928 |
| **Garrison, F. H.** | *History of Medicine* | 4th Ed. | (4) | 1929 |
| **Gillespie, R. D.** | *Hypochondria* | | | 1929 |
| **Green** | *Manual of Pathology* | 14th Ed. | (4) | 1928 |
| **Halliburton and MacDowall.** | *Handbook of Physiology* | 18th Ed. | (3) | 1928 |
| **Hare, H. A.** | *Use of Symptoms in the Diagnosis of Disease.* | 9th Ed. | (3) | 1928 |
| **Heberden** | *Introduction to the Study of Physic* | | (3) | 1929 |
| **Herrick, C. J.** | *Introduction to Neurology* | 4th Ed. | (3) | 1928 |
| **Hewer, E. E., and Sandes, G. M.** | *Introduction to the Study of the nervous System* | | | 1929 |
| **Heymans, C.** | *Le Sinus Carotidien* | | | 1929 |
| **Hotwell** | *A Poem* | | | |
| **Humphris, F. H.** | *Artificial Sunlight and its Therapeutic uses.* | 5th Ed. | | 1929 |
| **Hurst, A. F., and Stewart, M. J.** | *Gastric and Duodenal Ulcer* | | (3) | 1929 |
| **Jones, Sir R. and Lovett, R. W.** | *Orthopaedic Surgery* | 2nd Ed. | | 1929 |
| **Jorden, E.** | *Discourse of Naturall Bathes and Mineral Waters* | | | 1673 |
| **Joslin, E. P.** | *Treatment of Diabetes Mellitus.* | 4th Ed. | (3) | 1928 |
| **Joly, J. S.** | *Stone and Calculus Disease* | | (3) | 1929 |
| **Labat, G.** | *Regional Anæsthesia* | 2nd Ed. | (3) | 1928 |
| **Lockhart-Mummery, P.** | *After treatment of Operations* | 5th Ed. | (3) | 1929 |
| **Maclean, H.** | *Modern Views on Digestion and Gastric Disease* | 2nd Ed. | (3) | 1928 |
| **Maclean, H.** | *Modern Methods in the Diagnosis of Glycosuria and Diabetes* | 4th Ed. | (3) | 1927 |
| **Marshall, A. M.** | *The Frog* | 12th Ed. | (4) | 1928 |
| **May, C. H. and Worth C.** | *Manual of Diseases of the Eye* | 5th Ed. | (3) | 1927 |
| **Mead, S. V.** | *Diseases of the Mouth* | 3rd Ed. | (3) | 1928 |
| **Muir, R.** | *Textbook of Pathology* | 2nd Ed. | (4) | 1929 |
| **Myers, C. S.** | *Industrial Psychology* | | (4) | 1929 |
| *Nouveau Traité de médecine* Vol. XVII. | | | | 1929 |
| **Parfitt, J. B.** | *Operative Dental Surgery* | 2nd Ed. | (3) | 1923 |
| **Paterson, D., and Smith, J. F.** | *Feeding in Infancy and Childhood.* | 2nd Ed. | (3) | 1929 |
| **Pavlov, I. V.** | *Lectures on Conditioned reflexes* | | (4) | 1929 |
| **Pearson, W. J. and Wylie, W. G.** | *Recent Advances in Diseases of Children* | | (3) | 1928 |
| **Price, F. W.** | *Textbook of the Practice of Medicine* | 3rd Ed. | | 1929 |

| | | | | |
|---|---|---|---|---|
| **Pryde, J.** .. .. .. | *Recent Advances in Bio-chemistry* | 2nd Ed. | (3) | 1928 |
| *Radium Treatment of Cancer of the Uterus* .. .. .. .. .. | | 2nd Ed. | | 1929 |
| **Ramsay, E. M.** .. | *The Eye in General Medicine* .. | 2nd Ed. | (3) | 1929 |
| **Rolleston, Sir H. and McNee, J. W.** | *Diseases of the Liver, &c.* | 3rd Ed. | (3) | 1928 |
| **Rolleston, J. D.** | *Acute Infectious Diseases* .. .. .. | 2nd Ed. | | 1929 |
| **Ross, J. S., and Fairlie, H. P.** | *Handbook of Anæsthesia* .. | 3rd Ed. | (3) | 1929 |
| **Shaw, T. B.** .. .. | *Naval Hygiene* .. .. .. .. .. .. .. .. .. .. .. | | | 1929 |
| **Stander, H. G.** .. .. | *Toxemias of Pregnancy* .. .. .. .. .. .. | | (3) | 1929 |
| **Starling** .. .. .. | *Principles of Human Physiology* .. | 5th Ed. | (4) | 1930 |
| **Stitt, E. R.** .. .. | *Diagnosis and Treatment of Tropical Diseases.* | 5th Ed. | (3) | 1929 |
| **Sutherland, A.** .. .. | *The Nature and Qualities of Bristol Water* .. | | | 1758 |
| **Thursfield, H., and Paterson, D.** | *Diseases of Children* .. | 2nd Ed. | (3) | 1929 |
| **Tredgold, A. F.** .. | *Mental Deficiency* .. .. .. .. .. | 2nd Ed. | (3) | 1929 |
| **Treves-Barber, T. H.** | *Treatment of Varicose Veins* .. .. .. .. .. | | | 1929 |
| **Voronoff, S.** .. .. | *Testicular Grafting from Ape to Man* .. .. | | | N.D. |
| **Wheeler, Sir W. J. de. C.** | *Handbook of Operative Surgery* | 4th Ed. | (3) | 1925 |
| **Willich** .. .. .. | *The Hotwell Water, Bristol* .. .. .. .. .. | | | 1798 |
| **Worth, C.** .. .. .. | *Squint* .. .. .. .. .. .. .. .. .. .. | 6th Ed. | (3) | 1929 |
| **Wright, S.** .. .. .. | *Applied Physiology* .. .. .. .. .. .. | 3rd Ed. | | 1929 |
| **Yeomans, F. C.** .. | *Proctology* .. .. .. .. .. .. .. .. .. .. .. .. | | (3) | 1929 |

### TRANSACTIONS, REPORTS, JOURNALS, ETC.

| | | |
|---|---|---|
| *Annales de l'Institut Pasteur. Table Generale.* Vols. I.–XL. (1887-1927) | | 1929 |
| Annual Report of the Rockefeller Foundation, 1928 .. .. .. .. .. | (5) | 1929 |
| Annual Report of the Smithsonian Institution, 1928 .. .. .. .. .. | | 1929 |
| British Journal of Urology. Vol. I. and in continuation .. .. | (3) | 1929 |
| Burdett's Hospitals and Charities, 1930 .. .. .. .. .. .. .. .. .. | | 1930 |
| *Congres International de Médecine Tropical, etc.* Tomes I. and II., 1928 | (6) | 1929 |
| Far Eastern Association of Tropical Medicine. 7th Congress. Vol. 3 .. | (2) | 1927 |
| Medical Directory, 1930 .. .. .. .. .. .. .. .. .. .. .. .. .. .. .. | | 1930 |
| Methods and Problems of Medical Education. 15th Series .. .. | (5) | 1929 |
| Progressive Medicine, December, 1929 .. .. .. .. .. .. .. .. .. .. | | 1929 |
| Reports of St. Thomas's Hospital. Vol. 51 .. .. .. .. .. .. .. .. | | 1927 |
| Registrar-General's Decennial Supplement, 1921 .. .. .. .. .. .. | | 1927 |
| Surgical Clinics of North America, December, 1929 and February, 1930 | | 1929-30 |
| Transactions of the Association of American Physicians. Vol. 44 .. | | 1929 |
| Transactions of the Ophthalmological Society. Vol. 46 .. .. .. .. .. | | 1929 |
| Westminster Hospital Reports. Vol. 20 .. .. .. .. .. .. .. .. .. | | 1929 |

### Local Medical Notes

EXAMINATION RESULTS.

**University of Bristol.** Students of the University have recently passed the following examinations :—

M.D.—W. A. Gornall.

M.B., Ch.B.—*Second Examination.* (*Part I.*). *Pass :* Grace J. V. Ball, Dorothy E. Barber, A. G. W. Branch, J. R. G. Damrel, Rosalind M. S. Derham, N. H. Greenberg, G. L. L. Gurney, Irene G. Hamilton, J. N. Heales, Aldyth D. Jones, A. J. Matheson, A. C. Molden, C. H. G. Price, C. F. Rivington, S. Roberts, K. C. P. Smith, T. H. Taylor, Marion L. Tryon, F. L. Wollaston. *In Organic Chemistry* (*Completing Examination*) *:* Mary G. Thomas, S. P. N. Williams. *In Elementary Anatomy only :* T. R. V. Gurney.

M.B., Ch.B.—*Second Examination.* (*Part II.*). *Pass :* S. B. Adams, A. J. Board, Violet Fry (with Distinction in Anatomy), Margaret A. W. Hawkes, Winifred M. Hill, H. E. Pearse (with Distinction in Anatomy), Francis E. Powell.

M.B., Ch.B.—*Final Examination.* (*Part I.*) (*including Forensic Medicine and Toxicology*). *Pass :* J. S. Adamson, T. A. Barnabas, J. E. Brown, H. L. Cleave (with Distinction in Forensic Medicine and Toxicology), J. L. S. Coulter (with Distinction in Materia Medica, Pharmacy, Pharmacology and Therapeutics), C. J. N. Davis, F. W. A. Fosbery, L. R. Jordon (with Distinction in Pathology), J. A. Kersley, R. H. Moore, Elizabeth S. G. Owen, C. N. Royal, W. A. F. Taylor. *In Part I. only :* L. P. Ashton.

M.B., Ch.B.—*Final Examination.* (*Part II.*). *Pass :* (with Second Class Honours), J. J. J. Giraldi. *Pass :* J. E. Newton, N. C. Tanner. *In Group I.* (*Completing Examination*)*:* N. L. Price. *In Group II.* (*Completing Examination*) *:* N. D. Gerrish, Mabel F. Potter. *In Group II. only :* R. M. Norman.

L.D.S.—*First Professional Examination. Pass in Dental Anatomy* (*Completing Examination*) *:* Helena C. L. Blinkworth, G. N. Minifie. *In Anatomy and Physiology only :* R. L. Royal.

L.D.S.—*Second Professional Examination. Pass* (*in Dental Mechanics and Dental Metallurgy, Completing Examination*) *:* N. Miller, E. V. O'Hara, H. L. Saunders.

L.D.S.—*Final Professional Examination. Pass :* J. D. Galloway.

**Conjoint Board.**

M.R.C.S., L.R.C.P.—*Pass* (*in Physics*) *:* Miss I. G. Hamilton. (*In Medicine*) *:* G. F. Langley,* J. E. Newton,* S. C. Wake.* (*In Surgery*) *:* S. C. Wake,* U. M. Hopkins. (*In Midwifery*) *:* S. C. Wake,* H. M. Strover, K. I. Nicholls, U. M. Hopkins, J. L. S. James, H. F. G. Corfield.

L.D.S., R.C.S.—*First Professional Examination. Pass : Part I.* (*Dental Mechanics*) *:* O. F. Harley. *Part II.* (*Dental Metallurgy*) *:* O. F. Harley, W. A. Duly, A. E. Hawkins. *Part III.* (*a*) (*Anatomy and Physiology*) *:* A. E. Hawkins, C. A. J. Evans. *Part III.* (*b*) (*Dental Anatomy and Physiology*) *:* R. H. Green, H. W. South.

L.D.S., R.C.S.—*Final Examination. Part I. Pass :* E. S. Edwards, W. F. G. Harvey, J. J. Hinton, W. R. R. Mewton, K. V. M. Taylor-Milton, J. H. White.

---

The Bristol Medical Dramatic Club held their forty-seventh season on Friday and Saturday, March 14th and 15th. The play they had selected was "The Fourth Wall," by A. A. Milne, an excellent choice. The actors appeared all thoroughly at home in their parts, and gave a performance the vivacity and finish of which could not have been bettered. Their work, in fact, is without exaggeration of first class professional standard. It is difficult to select for praise any one name; there were no "weak places." Miss Collinson as "Susan Cunningham," on whom the whole of the last act turns, was magnificent. Mr. Denning as "Edward Carter" showed well how a man "can smile and be a villain." Mr. Bulleid and Mr. Lattey as the policeman father and detective son were particularly good—a little over-acting here would have produced a farce. The other members of the cast played up in a most generous and happy manner to give the effect of the best show the Medicals have ever put on.

* Qualified.

### Bristol Medico-Chirurgical Society.

---

### President.

C. FERRIER WALTERS, F.R.C.S.

### President-Elect.

D. C. RAYNER, F.R.C.S.

### Committee.

*Ex-Officio* { J. A. NIXON, *C.M.G.*, B.A., M.D. Cantab. (Editor of *Journal*).

W. A. SMITH, M.A., M.B. Oxon. (Hon. Librarian).

*Elected.*

A. L. FLEMMING, M.B., Ch.B. Brist. } Ex-Presidents.

W. K. WILLS, *O.B.E.*, M.A., M.B., B.Ch. Cantab. } Ex-Presidents.

H. L. ORMEROD, M.D., B.Ch., R.U.I. } Ex-Presidents.

DUNCAN WOOD, F.R.C.S.

R. S. STATHAM, *O.B.E.*, M.D., M.Ch. Brist.

H. CHITTY, M.S., F.R.C.S.

C. H. TERRY, M.A., M.B., B.Ch. Oxon.

E. W. HEY GROVES, M.S., F.R.C.S., B.Sc.

H. G. KYLE, M.A., M.D., B.Ch. Oxon.

### Honorary Secretary.

W. A. JACKMAN, M.B., F.R.C.S. Ed.

### Honorary Treasurer.

A. E. ILES, *O.B.E.*, M.B., B.S., F.R.C.S.

### University Library Sub-Committee.

W. A. SMITH, M.B., M.A. Oxon. G. PARKER, M.D., M.A. Cantab.

A. R. SHORT, M.D.

Medical Practitioners wishing to join the Society are requested to communicate with the Hon. Secretary, Mr. W. A. JACKMAN, 15 Mortimer Road, Clifton. The Annual Subscription is £1 11s. 6d., payable to Hon. Treasurer.

---

*Extracts from Journal By-laws.*

4.—The *Journal* shall be delivered free to Subscribers and also to Members of the Society whose subscriptions are not in arrear.

6.—The Annual Subscription to the *Journal* is 10/6, payable to Hon. Treasurer.

---

Communications intended for insertion in the *Journal* should be sent to the Editor, Dr. J. A. NIXON, 7 Lansdown Place, Clifton, Bristol.

Books for Review and Exchange Journals should be sent to the Assistant-Editor, *Bristol Medico-Chirurgical Journal*, Medical Library, The University, Bristol.

Communications referring to the delivery of the *Journal* should be sent to the Editorial Secretary, A. L. Flemming, 48 Pembroke Road, Clifton, Bristol.

Cases for Binding Vols. I—XLVI are now ready, and may be obtained, price 1/9 each (postage extra), upon application to J. W. ARROWSMITH LTD., Quay Street, Bristol; or the *Journal* will be bound, including Case, for 7/- per volume.

### Bristol Medico-Chirurgical Society.

### PAST PRESIDENTS.

| | |
|---|---|
| 1874—75. | FREDERICK BRITTAN, M.D., B.A. Dub. |
| 1875—76. | ROBERT WILLIAM COE. |
| 1876—77. | SAMUEL MARTYN, M.D. Ed. |
| 1877—78. | AUGUSTIN PRICHARD, M.D. Berlin. |
| 1878—79. | HENRY EDWARD FRIPP, M.D. Würzburg. |
| 1879—80. | WILLIAM MICHELL CLARKE. |
| 1880—81. | JOSEPH GRIFFITHS SWAYNE, M.D. Lond. |
| 1881—82. | EDWARD LONG FOX, M.D. Oxon. |
| 1882—83. | JAMES GEORGE DAVEY, M.D. St. And. |
| 1883—84. | WILLIAM JOHNSTONE FYFFE, M.D., B.A. Dub. |
| 1884—85. | GEORGE FORSTER BURDER, M.D. Aberd. |
| 1885—86. | WILLIAM HENRY SPENCER, M.D., M.A. Cantab. |
| 1886—87. | FRANCIS POOLE LANSDOWN. |
| 1887—88. | LEMUEL MATTHEWS GRIFFITHS. |
| 1888—89. | ROBERT SHINGLETON SMITH, M.D., B.Sc. Lond. |
| 1889—90. | NELSON CONGREVE DOBSON, Ch.M. Brist. |
| 1890—91. | SAMUEL HENRY SWAYNE. |
| 1891—92. | FRANCIS RICHARDSON CROSS, M.B. Lond., LL.D. Brist. |
| 1892—93. | EDWARD MARKHAM SKERRITT, M.D., B.S., B.A. Lond. |
| 1893—94. | JAMES GREIG SMITH, M.B., C.M., M.A. Aberd., F.R.S.E. |
| 1894—95. | ALFRED JAMES HARRISON, M.B. Lond. |
| 1895—96. | ARTHUR WILLIAM PRICHARD. |
| 1896—97. | ALFRED EDWARD AUST LAWRENCE, M.D., C.M. Aberd. |
| 1897—98. | JOHN EDWARD SHAW, M.B., C.M. Ed. |
| 1898—99. | ROBERT ROXBURGH, M.B. Ed. |
| 1899—00. | WILLIAM HENRY HARSANT. |
| 1900—01. | DAVID SAMUEL DAVIES, M.D. Lond. |
| 1901—02. | BARCLAY JOSIAH BARON, M.B., C.M. Ed. |
| 1902—03. | GEORGE MUNRO SMITH, M.D. Brist. |
| 1903—04. | JAMES PAUL BUSH, C.M.G., C.B.E., Ch.M. Brist. |
| 1904—05. | REGINALD EAGER, M.D. Lond. |
| 1905—06. | JOHN DACRE. |
| 1906—07. | JAMES TAYLOR. |
| 1907—08. | HENRY WALDO, M.D., C.M. Aberd. |
| 1908—09. | JOHN MICHELL CLARKE, M.D., M.A. Cantab. |
| 1909—10. | JAMES SWAIN, C.B., C.B.E., M.D., M.S. Lond. |
| 1910—11. | BERTRAM MITFORD HERON ROGERS, M.D., B.Ch., B.A. Oxon. |
| 1911—12. | CHARLES ALEXANDER MORTON. |
| 1912—13. | WALTER CARLESS SWAYNE, M.D., B.S. Lond. and Brist. |
| 1913—14. | PATRICK WATSON-WILLIAMS, M.D. Lond. |
| 1914—15. | WILLIAM HARRY CHRISTOPHER NEWNHAM, M.A., M.B. Cantab. |
| 1915—16 | GEORGE PARKER, M.A., M.D. Cantab. |
| 1916—17 | FRANCIS HENRY EDGEWORTH, M.A., M.D. Cantab., D.Sc. Lond. |
| 1917—18 | FREDERICK LACE. |
| 1918—19 | ROBERT GUTHRIE POOLE LANSDOWN, M.D., B.S. Durh. |
| 1919—20 | I. WALKER HALL, M.D., Ch.B. Vict. |
| 1920—21 | LEOPOLD ERNEST VALENTINE EVERY-CLAYTON, M.D. Lond. |
| 1921—22 | CYRIL HUTCHINSON WALKER, M.B., B.A. Cantab. |
| 1922—23 | JOHN ALEXANDER NIXON, C.M.G., B.A., M.D. Cantab. |
| 1923—24 | JOHN LACY FIRTH, M.D., M.S Lond. |
| 1924—25 | JOHN ODERY SYMES, M.D. Lond. |
| 1925—26 | THOMAS CARWARDINE, M.S. Lond. |
| 1926—27 | ARTHUR LAUNCELOT FLEMMING, M.B., Ch.B. Brist. |
| 1927—28 | W. KENNETH WILLS, M.A., M.B., B.Ch. Cantab. |
| 1928—29 | H. L. ORMEROD, M.D., B.Ch., R.U.I. |

1930.

### LIST OF SUBSCRIBERS

TO THE

### Bristol Medico-Chirurgical Journal.

### HONORARY MEMBERS OF THE SOCIETY.

| | |
|---|---|
| SWAIN, JAMES, *C.B.*, *C.B.E.*, M.D., M.S. .. .. .. .. .. | Wyndham House, Easton-in-Gordano. |
| WATSON-WILLIAMS, P., M.D. .. | 2 Rodney Place, Clifton. |
| PAUL BUSH, J., *C.M.G.*, Ch.M. | The Clifton Club. |

### SUBSCRIBERS.

*Those marked * are Members of the Society.*

| | |
|---|---|
| *ADAMS, A. W., M.S. Lond. ... ... ... ... ... | Rodney House, Clifton. |
| ADYE, W. J. H. ... ... ... ... ... ... ... ... | St. Margaret's, Bradford-on-Avon. |
| *ALDWINCKLE, H. M. M.B., Ch.B. Brist. ... ... | 30 E Berkeley Square, Clifton. |
| *ALEXANDER, D. A., M.B., Ch.B. Vict. ... ... | 112 Pembroke Road, Clifton, Bristol. |
| ALEXANDER, G. L., B.A. Cantab. ... ... ... | Principal Medical Officer, Freetown, Sierra Leone. |
| *ALFORD, A. F., M.B., Ch.B. Brist. ... ... ... | "Drofla," Kellaway Avenue, Westbury Park, Bristol. |
| ALFORD, H. J., M.D. ... ... ... ... ... ... ... | 4 Park Terrace, Taunton. |
| *ASKINS, R. A., M.D., B.Ch., B.A.O. Dub. ... ... | Guildhall, Bristol. |
| *AUBREY, T., M.B. Lond. ... ... ... ... ... ... | Bitton, near Bristol. |
| *BAKER, LILY A., M.D., B.Ch., B.A.O., R.U.I. ... | Vyvyan House, Clifton. |
| *BARBER, M. C., M.B., Ch.B. Brist. ... ... ... | Ingledene, High Street, Staple Hill, Bristol. |
| *BARKER, G. H., M.D., B.Sc. Lond. ... ... ... | 124 Redland Road, Bristol. |
| *BARTHOLOMEW, E. U. ... ... ... ... ... ... ... | 12 Lansdown Place, Clifton. |
| *BATT, J. C., M.B., Ch.B. Brist. ... ... ... | Bristol General Hospital. |
| BEATSON, D. H., M.B., Ch.B. ... ... ... ... | 8 Richmond Park Road, Clifton. |
| *BENSON, ELIZABETH, M.B., Ch.B. Brist. ... ... | 8 Theresa Place, Bristol Road, Gloucester. |
| *BERGIN, F. G. ... ... ... ... ... ... ... ... | 31 Oakfield Road, Clifton. |
| BERNARD, C. ... ... ... ... ... ... ... ... ... | 564 Fishponds Road, Fishponds, Bristol. |
| *BERRILL, T. H., M.B., Ch.B. Brist. ... ... ... | Bristol Royal Infirmary. |
| BERRY, F. C., M.D., B.Ch., B.A. Dub. ... ... | Locarno, Burnham, Somerset. |
| *BERRY, J. A., M.D. ... ... ... ... ... ... | Stoke Park Colony, Bristol. |
| *BIRRELL, J. A., M.D., M.S. Lond. ... ... ... | 2 Dundonald Road, Redland, Bristol. |
| *BLACHFORD, J. V., *C.B.E.*, M.D. Durh. ... ... | Milverton House, Long Ashton. |
| BLACKETT, J. F., M.D., B.S. Lond. ... ... ... | Hamsterley, Bloomfield Park, Bath. |
| *BLAGG, ARTHUR F., M.D. Durh.... ... ... ... | 6 Upper Belgrave Road, Clifton. |
| *BLATHWAYT, A. de V. ... ... ... ... ... ... | 6 Brock Street, Bath. |
| BLETCHLY, G. PLAYNE, M.B. Lond. ... ... ... | Hazelwood, Nailsworth, Glos. |
| *BODMAN, A. P., M.B., Ch.B. ... ... ... ... | 11 Hurle Crescent, Clifton, Bristol. |
| *BODMAN, C. O., M.D. Durh. ... ... ... ... | 17 Upper Belgrave Road, Clifton. |
| *BODMAN, F. H., M.B., Ch.B. Brist. ... ... ... | 2 Redland Court Road, Bristol. |
| *BODMAN, J. HERVEY, M.D. Lond. ... ... ... | 11 Hurle Crescent, Clifton, Bristol. |
| *BOULTON, B. J., M.B., Ch.B. Brist. ... ... ... | Ham Green Hospital. |
| *BRADBEER, E. G., M.B., Ch.B. Brist. ... ... | 32 Linden Road, Westbury Park, Bristol. |
| *BRADLEY, W. H., B.M., B.Ch. Oxon. ... ... | St. Wulstan's, Stratton-on-Fosse, Bath. |
| *BRAY, G., Lt.-Col *D.S.O.* ... ... ... ... ... | 46 Henleaze Avenue, Westbury-on-Trym. |
| *BRENTNALL, T. C. ... ... ... ... ... ... ... | Bellfield, Brightlingsea, Essex. |
| *BRIERLEY, C. E. ... ... ... ... ... ... ... | Bristol Royal Infirmary. |
| *BRISTOWE, H. C., M.D. Lond. ... ... ... ... | Wrington, Somerset. |

| | |
|---|---|
| *Brinckman, W. P., M.B., Ch.B. Brist. | 4 Oakland Road, Redland, Bristol. |
| *Brittain, B. A., M.B., Ch.B. Dub. | Bristol Royal Infirmary. |
| *Britton, R. B., M.B., B.S. Lond. | Troy House, Kingswood, Bristol. |
| *Buckmaster, G. A., M.A., M.D. Oxon. | University of Bristol. |
| *Burdon-Cooper, J., M.D., B.Sc. Durh. | 12 The Circus, Bath. |
| *Burges, R. | Druid's Mead, Stoke Bishop, Bristol. |
| *Burges, C. P., M.B., Ch B., | Ham Green Hospital, Pill, Nr. Bristol. |
| *Burgess, N. | Hereford House, Clifton. |
| *Burnett, R. H., M.B., B.S. Durh. | 479 Bath Road, Brislington, Bristol. |
| *Bush, G. B., M.B., Ch.B. Brist. | 5 Lansdown Place, Clifton. |
| *Cairns, F. J. | Devon House, Whitehall Road, Bristol. |
| *Carey, R. S., *O.B.E.*, B.A. Cantab. | 502 Bath Road, Brislington, Bristol. |
| *Carleton, H. H., M.A., M.D., B.Ch. Oxon. | 1 Lansdown Road, Clifton. |
| *Carling, A., M.A., M.B., B.C. Cantab. | 12 Great George Street, Bristol. |
| *Carter, T. M., *O.B.E.*, M.D. | 19 Westfield Park, Redland, Bristol. |
| *Carwardine, Thomas, M.B., M.S. Lond. | 16 Victoria Square, Clifton. |
| *Casson, E., M.B., Ch. B. Brist. | Dorset House, Clifton Down, Bristol. |
| *Cates, H. J., M.D. Lond. | Northwoods House, Winterbourne, Bristol. |
| *Cave, Edward J., M.D. Lond. | 16 The Circus, Bath. |
| *Chambers, E. R. | 9 Clifton Park, Clifton. |
| *Charles, J. R., M.A., M.D., B.C. Cantab. | 1 Victoria Square, Clifton. |
| *Chesterman, J. T., M.D., B.S. | Glen Avon, Broomfield Road, Bath. |
| *Chitty, H., M.S. Lond. | 46 Pembroke Road, Clifton. |
| *Christofferson, P. E., M.B., Ch.B. Brist. | 8 Lansdown Place, Clifton. |
| *Clarke, R. C., *O.B.E.*, M.B., Ch.B. Brist. | 29 Victoria Square, Clifton. |
| *Coates, Vincent M., *M.C.*, M.A., M.D., B.C. Cantab. | 10 The Circus, Bath. |
| Connellan, P. S. | 12 Bloomsbury Street, London, W.1. |
| *Cookson, R. G. | 35 Pembroke Road, Bristol. |
| *Coombs, Carey, M.D. Lond. | 3 Pembroke Road, Clifton. |
| *Cooper, William Russell | 13 Westfield Park, Redland, Bristol. |
| *Corfield, C., M.D. Durh. | 5 Kensington Place, Clifton. |
| *Cornall, A. F. M. | Carnarvon Lodge, Redland, Bristol. |
| *Cory, R. A. S. | Bristol Royal Infirmary. |
| *Cossham, W. L., M.B., Ch.B. Brist. | 32 Somerville Road, St. Andrew's Park, Bristol. |
| *Courtney, R. M., M.B. | 487 Gloucester Road, Bristol. |
| *Coxon, Beatrice | 16 Richmond Hill, Clifton. |
| Cridland, A. B. | Salisbury House, Chapel Ash, Wolverhampton. |
| *Cross, R. F., M.B. Lond., LL.D. Brist. | Worcester House, Clifton. |
| *Crossman, F. W., M.B. Durh. | White's Hill, Hambrook, near Bristol. |
| *Dacre, John | 14 Eaton Crescent, Clifton. |
| *Datta, S. S. M.B., Ch.B. Brist. | Snowdon House, Fishponds, Bristol. |
| *Davies, E. D. D. | Standish House, Stonehouse, Glos. |
| Devine, H., *O.B.E.*, M.D. Lond. | Holloway Sanatorium, Virginia Water. |
| *Dawe, J. H., M.B. Ed. | Lynwood House, Bickford Road, Bath. |
| *Dix, C. | 11 Victoria Square, Clifton. |
| *Dixon, Helen M., M.B., B.Ch. Brist. | 10 Whatley Road, Clifton. |
| *Dixon, T. B. | 11 Pembroke Road, Clifton, Bristol. |
| *Drapes, T. L., M.B., B.C., B.A. Cantab. | St. Maur, Beaufort Square, Chepstow. |
| *Duerden, J. R., M.B., Ch.B. Brist. | 149 Bell Hill Road, St. George, Bristol. |
| *Dutton, Hilda R. | 405 Stapleton Road, Bristol. |
| *Edgeworth, F. H., M.D., B.C., M.A. Cantab., D.Sc. Lond. | 13 Richmond Hill, Clifton. |
| *Eglinton, J. H. C. | Street, Somerset. |
| *Elkington, G. W., M.B., B.S. Lond. | Bruton, Somerset. |
| Elliot Bros, Ltd. | 20–22 O'Connell Street, Sydney, N.S.W., Australia. |
| Elliott, F. Percy, M.B., C.M. Aberd. | 113 Grove Rd., Walthamstow, London, E.17. |

| | |
|---|---|
| *Elliott, W. H. A., M.B., B.S. Lond. ... ... | 116 Pembroke Road, Clifton. |
| *Every-Clayton, L. E. V., M.D. Lond. ... ... | "Riverbank," Sunbury-on-Thames, Middlesex. |
| *Faill, C. J. C. ... ... ... ... ... ... ... ... | 19 Portland Square, Bristol. |
| *Fallon, Col. J. ... ... ... ... ... ... ... ... | 35 Henleaze Gardens, Henleaze, Bristol. |
| *Faraker, E. C. ... ... ... ... ... ... ... ... | Keinton Mandeville, Somerset. |
| Farnfield, W. W. ... ... ... ... ... ... ... | Clive Vale, Gillingham, Dorset. |
| *Fawn, G. F. ... ... ... ... ... ... ... ... ... | 6 Oakfield Road, Clifton. |
| *Fayle, B. W. D., M.B., Ch.B. ... ... ... ... | Reine Barnes, Dursley, Glos. |
| *Fells, A., M.B., C.M. Ed. ... ... ... ... ... | 6 Elton Road, Clifton. |
| *Fells, G. R., M.B., Ch.B. Brist. ... ... ... | Bristol General Hospital. |
| *Fells, R. R., M.B., B.S. Lond. ... ... ... | 12*d* Cotham Road, Bristol. |
| *Firth, J. L., M.D., M.S. Lond. ... ... ... | 8 Victoria Square, Clifton. |
| *Fisher, A. C. ... ... ... ... ... ... ... ... | Bristol Royal Infirmary. |
| *Flemming, A. L., M.B., Ch.B. Brist. ... ... | 48 Pembroke Road, Clifton. |
| *Flemming, C. E. S. ... ... ... ... ... ... ... | Manvers House, Bradford-on-Avon. |
| *Forrester-Brown, Maud, M.D., M.S. ... ... | 22 Coombe Park, Bath. |
| *Foss, E. V., M.D., Ch.B. Brist. ... ... ... | Summer Hill House, St. George, Bristol. |
| *Fox, F. E., B.A. Camb. ... ... ... ... ... | Brislington House, Brislington, Bristol. |
| *Fraser, A. D., M.A., M.B., Ch.B. Glasg. ... | 4 Alexandra Road, Clifton. |
| *Freeth, H., M.D. ... ... ... ... ... ... ... ... | 93 Redland Road, Bristol. |
| *Fuller, H. L. ... ... ... ... ... ... ... ... | 9 Gay Street, Bath. |
| *Garden, R. R., M.A., M.B., B.Ch. ... ... ... | 9 Harley Place, Clifton. |
| Garland, E. B., M.B., C.M. Ed. ... ... ... | 114*a* Hampton Road, Redland, Bristol. |
| *Gee, C. A. H., M.B., Ch.B. Brist. ... ... ... | Portbury, Nr. Bristol. |
| *Gibbs, A. N. Godby ... ... ... ... ... ... ... | 30 Whiteladies Road, Clifton. |
| *Giraldi, J. J., M.B., Ch.B. Brist. ... ... ... | Bristol General Hospital. |
| *Glenny, E. T., M.B., B.S. Lond. ... ... ... | 102 Pembroke Road, Clifton. |
| *Gordon, R. G., M.D., B.Sc. Ed. ... ... ... | 9 The Circus, Bath. |
| *Gorham, A. P., M.B., Ch.B. ... ... ... ... | 53 Westbury Road, Westbury-on-Trym. |
| *Gornall, W. A., M.B., Ch.B. Brist. ... ... ... | 23 Windsor Road, St. Andrew's Park, Bristol. |
| *Green, T. A., *D.S.O.*, M.D., C.M. Ed. ... ... | 33*a* Whiteladies Road, Clifton. |
| *Greenlaw, Madge E., M.B., Ch.B. Brist. ... | 194 Redland Road, Bristol. |
| Griffiths, Cornelius A. ... ... ... ... ... | 35 Newport Road, Cardiff. |
| *Griffiths, J. S. ... ... ... ... ... ... ... ... | Redland Park House, Bristol. |
| *Griffiths, S. J. H., M.B., Ch.B. Brist. ... ... | Bristol General Hospital. |
| *Groves, E. W. Hey, M.D., M.S. Lond. ... ... | 24 Victoria Square, Clifton. |
| *Hadfield, G., M.D. Lond. ... ... ... ... ... | Royal Free Hospital, London. |
| *Hall, E. George, M.B. Lond. ... ... ... ... | 42 Apsley Road, Clifton, Bristol. |
| *Hall, I. Walker, M.D., Ch.B. Vict. ... ... | Shortwood Lodge, Pucklechurch, Glos. |
| *Ham, C. I., M.B., Ch.B. Brist. ... ... ... ... | 16 Elm Lane, Redland, Bristol. |
| *Harris, H. Elwin, B.A., M.B. Cantab. ... ... | 13 Lansdown Place, Clifton |
| *Harris, H. E., M.A., M.B., B.Ch. Cantab. ... | 13 Lansdown Place, Clifton. |
| *Harsant, W. H. ... ... ... ... ... ... ... ... | Tower House, Clifton Down Road, Clifton. |
| *Henderson, James, M.B., Ch.B. ... ... ... | Nordrach upon Mendip, Blagdon, Somerset. |
| *Hector, F., M.D. Lond. ... ... ... ... ... ... | Chelsea Hospital for Women, Arthur Street, Chelsea, S.W.3. |
| *Herapath, C. E. K., *M.C.*, M.D. Lond. ... ... | Ormlie, Clifton. |
| *Heron, Gordon, M.B., Ch.B. Brist. ... ... | 34 Gloucester Road, Bristol. |
| *Heron, Gordon, Mrs., M.B., Ch.B., Brist. | 34 Gloucester Road, Bristol. |
| *Hooker, J. A., M.B., Ch.B. ... ... ... ... | 16 St. Edyth's Road, Sea Mills. |
| *Hosken, J. G. F. ... ... ... ... ... ... ... ... | Uley, Glos. |
| Hospital, Bristol General (*Secretary*, T. W. Gregg). | |
| *Houghton, L. M., M.B., B.S. Lond. ... ... | 74 Church Road, Redfield, Bristol. |
| *Hughes, T. I. ... ... ... ... ... ... ... ... | Bristol General Hospital. |
| *Hughes F. W. Terrell, M.B., Ch.B. Brist. ... | Chew Magna, Somerset. |
| *Hughes, D., M.B., B.Ch., B.A.O., N.U.I. ... | 12*d* Cotham Road, Bristol. |

*Hunter, F. S. ... c/o Messrs. J. Wright & Sons Ltd., Publishers, Bristol.

*Iles, A. E., *O.B.E.*, M.B., B.S. Lond. ... 17 Victoria Square, Clifton.
Infirmary, Bristol Royal (*Secretary*, E. C. Smith).
Ingle, C. Durban ... Somerton, Somerset.

*Jackman, W. A., M.B., Ch.B. Brist. ... 15 Mortimer Road, Clifton.
*James, J. A., M.D. Lond. ... 5 Rodney Place, Clifton.
*James, A. D., M.B., Ch.B. Brist. ... Bristol Royal Infirmary.
*Jenkins, R. D. ... Bristol General Hospital.
*Jenkins, F. G., M.B., Ch.B. Brist. ... 31 Berkeley Square, Clifton, Bristol.
*Johnson, R. G., M.B. Lond. ... 119 Redland Road, Bristol.
*Joll, C. A., M.B., M.S. Lond. ... 64 Harley Street, London.
*Joscelyne, P. C., M.B., Ch.B. Brist. ... 70Longmead Avenue, Bishopston, Bristol.

*Kemm, N. F., M.B., B.S. Lond. ... 188 Redland Road.
*Kennedy, W. P., M.D. Dub. ... 3 Gay Street, Bath.
*Kerfoot, Stanley J. ... 194 Redland Road, Bristol.
*Kingston, S. H., M.B., Ch.B. Brist. ... 3 Whiteladies Road, Clifton.
*Kyle, H. G., M.A., M.D., B.Ch. Oxon. ... 31 Westbury Road, Bristol.

*Lace, F. ... 5 Gay Street, Bath.
*Lees, E. Leonard, M.D., C.M. Ed. ... 22 Richmond Hill, Clifton.
*Legat, R. Eddowes, M.B., C.M. Ed. ... Murray House, Staple Hill, Bristol.
Leigh, W. W. ... Llansamnor House, Cowbridge, Glam.
*Leonard, R. C., M.D. Durh. ... Bower Ashton, Bristol.
*Lester, Muriel A., M.B., B.S. ... 110 Avon Vale Road, Redfield, Bristol.
*Levis, J. S., *M.C.*, M.B., B.Ch., N.U.I. ... 20 Gay Street, Bath.
*Linton, Marion S., M.B. Lond. ... 21 Oakfield Road, Clifton.
*Lister, A. E. J., M.B., B.S. Lond. ... 86 Pembroke Road, Clifton.
*Lister, L. Margaret ... 12 All Saints Road, Clifton.
*Logan, H. B. ... Elmwood, Aberdeen Road, Clifton.
*Lucas, J. E. ... 48 Coronation Road, Bristol.
*Lucas, J. J. S., M.D. Lond. ... 186 Wells Road, Totterdown, Bristol.
*Lysaght, A. C. ... 8 Clifton Hill, Bristol.

*Macfie, J. D., M.B., Ch.B. ... Winsley Sanatorium, near Bath.
Mackinnon, Charles, M.B., C.M., M.A. Glasg. Davaar, Cirencester.
Maclean, Sir Ewen J., M.D., C.M. Ed. ... 12 Park Place, Cardiff.
*MacLeod, Donald, M.B., C.M. Ed. ... 84 Redland Road, Redland, Bristol.
*MacLeod, G., *M.C.*, M.D., Ch.B. ... "Wargrave," Clevedon.
*MacWatters, J. C., *M.B.E.* ... Almondsbury, near Bristol.
Marle, S. ... Newbridge Road, Bath.
Marshall, Arthur L., M.B., B.A. Cantab. ... Raveningham, Norwich.
Mather, J. S., M.B., C.M. Aberd. ... Lawrence Hill House, Bristol.
*Mathew, W. E., M.D., C.M. Toronto ... 85 Coldharbour Road, Redland.
*Mayes, F. J. A. ... 79 Pembroke Road, Clifton.
*McCrea, Phillip, M.D., B.Ch. ... 56 Kellaway Avenue, Bristol.
*McKenzie, W. G., *M.C.* ... 101 Coronation Road, Bristol.
*McLannahan, J. G. ... The Mount, Stonehouse.
Miall, C. L'O ... Elm Hayes, Paulton, near Bristol.
*Milling, T., M.B., Ch.B. Belfast ... 1 Vicarage Road, Southville.
*Milner, A., M.B., B.Ch. Dub. ... 61 Cotham Brow, Bristol.
*Moore, C. A., M.B., M.S. Lond. ... 56 St. Paul's Road, Clifton.
*Moore, L. A. ... Hillsborough, Cotham Park, Bristol.
*Morris, A. G. ... 18 Westbury Park, Westbury-on-Trym.
*Morris, L. N., M.B., Ch.B. Brist. ... 24 All Hallows Road, Upper Easton, Bristol.

*Mumford, W. G., M.B. Lond. ... 18 The Circus, Bath.
*Mundy, G. S., M.B., B.Ch. Brist. ... Homewood, Coombe Dingle, Bristol.
*Murphy, F. D., M.B., Ch.B., B.Sc. Cork ... Southmead Hospital, Bristol.
*Myles, G. T. ... 7 Apsley Road, Clifton.

| | |
|---|---|
| *Neild, Newman, M.B., B.Ch. Vict. ... ... ... | 9 Richmond Hill, Clifton. |
| *Newnham, W. H. C., M.B., M.A. Cantab. ... | 3 Lansdown Place, Victoria Square, Clifton. |
| *Nichols. F. C., *M.C.*, M.B., Ch.B. Brist. ... | 38 Whiteladies Road, Clifton. |
| *Nixon, J. A., *C.M.G.*, M.D. Cantab. ... ... | 7 Lansdown Place, Clifton. |
| *Noble, J. Innes, M.B., Ch.B. ... ... ... ... | 102 Pembroke Road, Clifton. |
| *Nott, Winifred, M.B., Ch.B. Brist.... ... ... | "Fassiefern," Upper Cranbrook Road, Redland, Bristol. |
| *Ormerod, H. Lawrence, M.D., B.Ch. R.U.I. ... | 2 Henleaze Road, Westbury-on-Trym, Bristol. |
| Orr-Ewing, H. J., *M.C.*, M.D. Lond. ... ... | English Mission Hospital, Jerusalem. |
| *Parker, George, M.D., M.A. Cantab. ... ... | 14 Pembroke Road, Clifton. |
| *Parkes, J. A., M.B., Ch.B. Liverpool ... ... | 8 South Road, Redland, Bristol. |
| Parkinson, Lt.-Col. G. S., *D.S.O.*, R.A.M.C. ... | R.A.M.C. Mess, Grosvenor Place, London, S.W.1. |
| *Parry, R. H., M.B., B.S. Lond. ... ... ... | Newby, Holmes Grove Road, Henleaze. |
| Parsons, Sir J. H., M.B., B.S., B.Sc. Lond. ... | 54 Queen Anne Street, London, W. |
| *Pearce, J. L., M.B., Ch.B. Ed. ... ... ... | Hambrook Court, near Bristol. |
| *Perry Bruce, M.B., Ch.B. ... ... ... ... | Bristol General Hospital. |
| *Phillips, P., M.B., Ch.B. Brist.... ... ... ... | Southmead Hospital, Bristol. |
| *Pinkerton, J. M. ... ... ... ... ... ... ... ... | 5 The Circus, Bath. |
| *Pollard, J., M.B., B.Ch., B.A.O. ... ... ... | 88 Coronation Road, Bristol. |
| *Powell, J. J., M.D. Lond. ... ... ... ... | 2 Gloucester Road, Bishopston, Bristol. |
| *Price, H. H., M.B., C.M. Ed. ... ... ... ... | 17 Oakfield Road, Clifton. |
| *Pring, C. E., L.R.C.P. & S. Ed. ... ... ... | 14 West Park, Clifton, Bristol. |
| *Prowse, D. C., M.B., Ch.B. Brist. ... ... ... | Wigmore House, Thornbury, nr. Bristol. |
| *Rayner, D. C. ... ... ... ... ... ... ... ... | 9 Lansdown Place, Clifton, Bristol. |
| *Renton, R. A. S., *M.C.*, M.D. Durh. ... ... | Emsworth House, Clevedon. |
| *Robertson, D., M.B., Ch.B. Ed. ... ... ... | 205 Wells Road, Knowle, Bristol. |
| *Rogers, B. M. H., M.D., B.Ch., B.A. Oxon. ... | 14 Mortimer Road, Clifton. |
| *Rogers, Herbert, M.B., Ch.B. ... ... ... | 11 Clifton Hill, Bristol. |
| *Rolfe, R., B.A., M.B., B.C. Cantab. ... ... | Park House, St. Andrew's Road, Avonmouth. |
| *Ross, C., M.D. ... ... ... ... ... ... ... ... | Bristol Royal Infirmary. |
| *Rowley, A. T. ... ... ... ... ... ... ... ... | Wrington. |
| *Salisbury, Walter, M.D., M.S. Lond. ... ... | Woodlands, 20 Billing Road, Northampton. |
| Savage, W. G., M.D., B.Sc. Lond. ... ... ... | "Bourn," 7 Trewartha Park, Weston-super-Mare. |
| *Scarff, G., M.B., Ch.B. ... ... ... ... ... | 5 Rodney Place, Clifton. |
| *Scott, H. H. ... ... ... ... ... ... ... ... ... | 78 Old Market Street, Bristol. |
| *Shaw, J. E., M.B., C.M. Ed. ... ... ... ... | 23 Caledonia Place, Clifton. |
| *Shepherd, H. L., M.B., Ch.M. Brist. ... ... | 16 Victoria Square, Clifton. |
| *Short, A. R., M.D. ... ... ... ... ... ... | 69 Pembroke Road, Clifton. |
| *Short, L. J., M.D., B.S. Lond., M.D. Brist. ... | Maywood, Atlantic Road South, Weston-super-Mare. |
| *Smith, G. Cockburn, M.D. Brux. ... ... ... | 12 South Road, Newton Abbot. |
| *Smith, T. Wilson ... ... ... ... ... ... ... | 26 The Circus, Bath. |
| *Smith, W. Alexander, M.B., M.A. Oxon. ... | 70 Pembroke Road, Clifton. |
| *Smythe, H. J. D., *M.C.*, M.B., M S. ... .. | 15 Eaton Crescent, Clifton. |
| *Somers, Mary, Miss ... ... ... ... ... ... | 2 Ashcombe Road, Weston-super-Mare. |
| *Spoor, A., M.B., B.Ch. Cantab. ... ... ... | The Hydro, Bristol. |
| *Stanger, G. ... ... ... ... ... ... ... ... ... | Olveston, Nr. Bristol. |
| *Staniland, M. F. ... ... ... ... ... ... ... ... | 255 Stapleton Road, Bristol. |
| *Statham, R. S. S., *O.B.E.*, M.D., M.Ch. Brist. | 10 Beaufort Road, Clifton, Bristol. |
| *Stewart, A. L., D.M., D.Ch. Brux. ... ... | 31 Howard Road, Southville, Bristol. |
| *Stock, W. S. V., M.B. Lond. ... ... ... ... | 1 Mortimer Road, Clifton. |
| *Strover, H. W. M., *O.B.E.*, M.B., Ch.B. Aberd. ... ... ... ... ... ... ... | 24 Gloucester Road, Bishopston, Bristol. |

| | |
|---|---|
| *SUTTON, G. E. F., M.B., B.S. Lond. ... ... ... | 21 Victoria Square, Clifton. |
| *SUTHERLAND, GORDON ... ... ... ... ... ... | 5 Rodney Place, Clifton, Bristol. |
| *SYMES, J. O., M.D. Loud. ... ... ... ... ... | 71 Pembroke Road, Clifton. |
| *TASKER, D. G. C., M.B., M.S. Lond. ... ... | 10 Christchurch Road, Clifton. |
| *TAYLOR, A. L., M.D. Lond.... ... ... ... ... | Bristol General Hospital. |
| TAYLOR, A. L.... ... ... ... ... ... ... ... | Bristol Royal Infirmary. |
| *TAYLOR, H. N., M.A. Cantab., M.D. Dub. ... | Hillside, Ebbw Vale. |
| *TEMPLE, G. H., M.B., C.M. Ed. ... ... ... | Ailanthus, Weston-super-Mare. |
| *TERRY, C. H., M.A., M.B., B.Ch. Oxon. ... ... | 15 The Circus, Bath. |
| THIN, JAMES ... ... ... ... ... ... ... ... | 54 & 55 South Bridge, Edinburgh. |
| *THOMPSON, J., M.B., Ch.B. ... ... ... ... ... | Eastbourne House, Devizes. |
| *THOMAS, J. D., M.B. Cantab. ... ... ... ... | Northwoods, Winterbourne. |
| *TODD, A. T., *O.B.E.*, M.B., Ch.B. Ed. ... ... | 34 Alma Road, Clifton. |
| *TRIPP, DOROTHY, M. H., M.B., Ch.B. Brist. ... | Harford House, Chew Magna, near Bristol. |
| *TRIST, J. R., R. *M.C.* ... ... ... ... ... | 120 Henleaze Road, Henleaze. |
| *TRYON, V., M.B., Ch.B. Brist. ... ... ... ... | 3 Harley Place, Clifton. |
| *VICKERY, W. H. ... ... ... ... ... ... ... ... | "Shirley," Broadoak Road, Weston-super-Mare. |
| *WALKER, CYRIL H., M.B., B.A. Cantab. ... ... | 8 Oakfield Road, Clifton. |
| *WALLACE, JOHN, *O.B.E.*, M.B., C.M. Ed. ... | 10 Knightstone Road, Weston-super-Mare. |
| *WALSH, M. J., M.B., Ch.B. Dub.... ... ... ... | 2 Knowle Road, Bristol. |
| *WALTERS, C. F. ... ... ... ... ... ... ... | 5 Mortimer Road, Clifton. |
| *WANKLYN-JAMES, C. W., *O.B.E.* ... ... ... | 3 Mortimer Road, Clifton. |
| *WARREN, R., M.A., M.D. Oxon. ... ... ... ... | Ellenborough Hall, Weston-super-Mare. |
| *WATERHOUSE, R., M.D. Lond. ... ... ... ... | 25 The Circus, Bath. |
| *WATSON-WILLIAMS, E., *M.C.*, B.A. Cantab., F.R.C.S. ... ... ... ... ... ... ... ... | 12 Victoria Square, Clifton. |
| *WESTON, B. A. A., M.B., B.Ch. Brist. ... ... | 30 Cotham Grove, Bristol. |
| *WEATHERHEAD, E., M.B., B.Ch. Cantab. ... | 115 Queen's Road, Clifton, Bristol. |
| WESTON, B. A., M.B. ... ... ... ... ... ... | Brookside, Admaston, Wellington Shropshire. |
| *WHELTON, A., B.A., M.B., Ch.B. Cork ... ... | 41 Guinea Street, Redcliffe, Bristol. |
| *WHICHER, A. H. ... ... ... ... ... ... ... | 115 Queen's Road, Clifton. |
| *WHITE, E. B. C. ... ... ... ... ... ... ... | Mental Hospital, Fishponds, Bristol. |
| WHITEHOUSE, H. BECKWITH, M.S. Lond. ... | 62 Hagley Road, Edgbaston, Birmingham. |
| WIGAN, C. A., M.D. Durh., J.P. ... ... ... | Deepdene, Portishead. |
| WIGMORE, J., M.D., R.U.I.... ... ... ... ... | 14 Catherine Place, Bath. |
| WILLCOX, R. LEWIS ... ... ... ... ... ... ... | 97 London Road, Salisbury. |
| WILLIAMS, S. R., M.B. Lond. ... ... ... ... | Buckfastleigh, Devon. |
| *WILLIAMS, A. R., M.B., Ch.B., Brist. ... ... | Bristol Royal Infirmary. |
| *WILLS, W. K., *O.B.E.*, M.B., B.C. Cantab. ... | 1 Victoria Square, Clifton. |
| *WOOD, DUNCAN ... ... ... ... ... ... ... | 5 Eaton Crescent, Clifton. |
| *WOOD, W. V., *M.C.*, B.A. Cantab. ... ... ... | Henley Lodge, Yatton, Som. |
| *WRIGHT, A. J., M.B., B.S. Lond. ... ... ... | Clifton Park, Clifton. |
| *ZEALAND, L., M.D. Man. ... ... ... ... ... | Brecon Lodge, Westbury-on-Trym, Bristol. |

### Publications Received.

From CASSELL AND CO. LTD.:

*Anæsthesia and Anæsthetics.* By F. S. ROOD, M.B., B.S. (Dunelm.), and H. N. WEBBER, M.A., B.Chir. (Cantab.). 1930. Price 14s. net.

From PAUL B. HOEBER:

*Radium in General Practice.* By A. JAMES LARKIN, B.Sc., M.D., D.N.B. 1929. Price $6.00 net.

From THE LANCET LTD.

*Guy's Hospital Reports.* Vol. 79, 4. 1929.

From H. K. LEWIS AND CO. LTD.:

*A New Treatment for Tuberculosis.* By PIERRE HULLIGER, M.D. 1930. Price 8s. 6d. net.

*Le Sinus carotidien et les autres zones vasosensibles réflexogènes.* Par le DOCTEUR CORNEILLE HEYMANS. 1929. Price 5s. net.

*Affections of the Eye in General Practice.* By R. LINDSAY REA, B.Sc., M.D., M.Ch., F.R.C.S. 1930. Price 10s. 6d. net.

*A Shorter Surgery.* By R. J. MCNEILL LOVE, M.B., M.S. (Lond.), F.R.C.S. (Eng.). Second Edition. 1930. Price 16s. net.

*Nasal Catarrh.* By W. STUART LOW, F.R.C.S. (Eng.). 1930. 5s. net.

*Recent Work on Colporrhaphy, Rheumatism and Coli-Bacilluria.* By E. HESKETH ROBERTS, F.R.C.S. (Edin.). Second Edition. 1930. Price 3s. 6d. net.

*Radium and Cancer (Curietherapy).* By D. C. L. FITZWILLIAMS, C.M.G., F.R.C.S. 1930. Price 12s. 6d. net.

From MACMILLAN AND CO. LTD.:

*The Sthenics.* By Sir JAMES K. FOWLER, K.C.V.O., C.M.G., M.A., M.D. 1930. Price 3s. 6d. net.

From NURSING MIRROR LTD.:

*Burdett's Hospitals and Charities.* Fortieth Year. 1930. Price 21s. net.

From OXFORD UNIVERSITY PRESS:

*Applied Physiology.* By SAMSON WRIGHT, M.D., M.R.C.P. Third Edition. 1929. Price 18s. net.

From JOHN WRIGHT AND SONS LTD.:

*British Journal of Surgery.* January, 1930. Price 12s. 6d. net.

*Physical Signs in Clinical Surgery.* By HAMILTON BAILEY, F.R.C.S. (Eng.). Second Edition. 1930. Price 21s. net.

*The Dramatic in Surgery.* By G. GORDON-TAYLOR, O.B.E., M.A., F.R.C.S. 1930. Price 12s. 6d. net.

*Through the States with a Seeing Eye.* By Prof. R. J. A. BERRY, M.D., F.R.C.S., F.R.S. (Edin.). 1930. Price 5/- net.

